# Illumina-based transcriptomic profiling of *Panax notoginseng* in response to arsenic stress

**DOI:** 10.1186/s40529-016-0128-8

**Published:** 2016-06-02

**Authors:** Yanfang Liu, Yanhua Mi, Jianhua Zhang, Qiwan Li, Lu Chen

**Affiliations:** Quality Standard and Testing Technology Research Institute, Yunnan Academy of Agricultural Sciences, No. 2238, Beijing Road, Kunming, 650205 People’s Republic of China

**Keywords:** *Panax notoginseng*, Arsenic stress, Illumina sequence, Differential expressed genes (DEGs)

## Abstract

**Background:**

*Panax notoginseng*, a famous herbal medicine, has recently attracted great attention on its safety and quality since *P*. *notoginseng* can accumulate and tolerate As from growing environment. For the purpose of understanding As damage to the quality of *P*. *notoginseng* as well as corresponding tolerance mechanisms, genes involved in As stress response were identified using Illumina sequencing.

**Results:**

Totally 91,979,946 clean reads were generated and were de novo assembled into 172,355 unigenes. A total of 81,575 unigenes were annotated in at least one database for their functions, accounting for 47.34 %. By comparative analysis, 1725 differentially expressed genes (DEGs, 763 up-regulated/962 down-regulated) were identified between As stressed plant (HAs) and control plant (CK), among which 20 DEGs were further validated by real-time quantitative PCR (qRT-PCR). In the upstream and downstream steps of biosynthesis pathways of ginsenosides and flavonoids, 7 genes encoding key enzymes were down-regulated in HAs. Such down-regulations were also revealed in pathway enrichment analysis. Genes encoding transporters (transporters of ABC, MATE, sugar, oligopeptide, nitrate), genes related to hormone metabolism (ethylene, ABA, cytokinin) and genes related to arsenic accumulation (HXT, NRAMP, MT and GRX) were differentially expressed. The up-regulated genes included those of oxidative stress-related protein (GSTs, thioredoxin), transcription factors (HSFs, MYBs) and molecular chaperones (HSP).

**Conclusions:**

The down-regulation of biosynthesis of ginsenoside and flavonoid indicated that As accumulation in *P*. *notoginseng* can cause not only safety hazard, but also qualitative losses. Aside from the results of arsenic content of seedling roots, the ability of *P. notoginseng* to over-accumulate arsenic can also be explained by the differential expression of genes of HXT, NRAMP, MT and GRX. To illustrate the detoxification mechanism of *P. notoginseng*, differential expression of genes encoding oxidative-related proteins, transcription factors, molecular chaperones, transporters and hormone were revealed in our study, which agreed with those reported in *Arabidopsis* to a certain extent, indicating *P*. *notoginseng* and *Arabidopsis* shared some common detoxification mechanisms in response to As stress. The longer As treatment in our study may account for the smaller quantity of related DEGs and smaller degree of expression differences of certain DEGs compared with those of *Arabidopsis*.

**Electronic supplementary material:**

The online version of this article (doi:10.1186/s40529-016-0128-8) contains supplementary material, which is available to authorized users.

## Background

*Panax notoginseng* (Burk.) F. H. Chen (Araliaceae) is a perennial herb and has been used as traditional Chinese medicine for thousands of years (Wang et al. 2013; Xia et al. 2014). Ginsenosides (also called as triterpene saponins) and flavonoid are known as the main pharmacologically active compounds found in *P*. *notoginseng* (Ng 2006; Sun et al. 2010). *P*. *notoginseng* has been widely consumed as home remedies in both raw (fresh or cooked) and processed (medicinal products) forms (Gong et al. 2015; Wang et al. 2012), and has been attracting more and more attention worldwide due to its anti-oxidative, anti-inflammatory, anti-coagulation, neuro-protective, anti-fibrotic, anti-diabetic, anti-cancer as well as anti-atherogenic effects (Ng 2006; Sun et al. 2010; Liu et al. 2014). Such increase in popularity has also brought concerns and fears over the quality and safety of the unprocessed products due to the increasing contamination of heavy metals through natural and anthropogenic pathway (Liu et al. 2013a, 2010).

*P*. *notoginseng* is native to southern China and primarily cultivated in Wenshan, Yunnan province of China, where it occupies 98 % of total yield (Guo et al. 2010; Guo 2007). However, soil in Wenshan has been partly contaminated by As owing to frequent mining activities and large-scale use of As containing pesticide. Different from most plants, toxicity threshold of arsenic to one-year-old seedlings of *P. notoginseng* is 13 mg kg^−1^ (Mi et al. 2015), while adult plants of *P. notoginseng* can exhibit great tolerance to highly As contaminated environment of up to 250 mg kg^−1^. Besides, the herb is capable of absorbing As, causing As concentration in root, stem and leaves exceeding the national standard (<2 mg kg^−1^) significantly (Yan et al. 2012, 2013). Furthermore, flavonoid content of *P*. *notoginseng* was reported to reduce along with the increase of As accumulation (Zu et al. 2014), which also contradicts with most plant species and genus (Su and Zhou 2009). Until now, influence of As stress to the quality of *P*. *notoginseng* and corresponding tolerance mechanism have been poorly understood.

Transcriptome sequencing provides a cost-effective means of qualitative and quantitative analyses of gene transcripts in many non-model species. In this study, we performed genome-wide transcriptome profiling to identify the genes of *P*. *notoginseng* responding to As stress to elucidate the quality changes and the tolerance mechanism of *P*. *notoginseng* in As contaminated conditions.

## Methods

### Plant material, treatment of As stress, RNA extraction and measurement of As content

Seeds of *P*. *notoginseng* were sown in trays for seedling nursing in January 2014. In October, fifty-two 10-cm-high healthy seedlings were chosen, including four seedlings bred from the same parent plant. After removing soil and rotten/injured roots, the 52 seedlings were transplanted to 500 mL glass jar filled with 150 mL nutrient solution (for *P*. *notoginseng*’s exclusive use) (Mi et al. 2015), making sure the roots immersed in solution and the stems aloft. After 14 days, seedlings were divided into two groups: (1) 26 seedlings for control (CK), (2) 26 seedlings for 14 days’ exposure experiment: 40 mg L^−1^ Na_2_HAsO_4_·7H_2_O (HAs). Roots of the 52 seedlings were afterwards rinsed thoroughly with deionized water. The four seedlings sharing the same parent plant were allocated to CK and HAs groups equally, among which, one root of CK and one root of HAs were for RNA extraction respectively. Total RNA of each sample was extracted using an E.Z.N.A. Plant RNA Kit (OMEGA Bio-Tec, USA). The quality, purity, concentration and integrity of the RNA samples were assessed accordingly. 3 μg RNA per sample was sent to Novogene Bioinformatics Technology CO., LTD, Beijing, China (http://www.novogene.cn) for Illumina sequencing, and the remained was for real-time quantitative PCR (qRT-PCR).

The rest 50 roots were for the purpose of As content comparison. Roots were oven dried at 95 °C for 30 min, later dried to constant weight at 56 °C (Mi et al. 2015). Dried roots were pounded into powder and 0.5 g powder was digested in HNO_3_/HClO_4_ (10 mL/1 mL) at room temperature for 24 h, then under gradually heating condition from 50 to 150 °C till white smoke turned up. After cooling, 0.5 mL HNO_3_ and deionized water was added to constant volume of 25 mL (Qiang et al. 2003). Arsenic concentration was determined by inductively coupled plasma mass spectrometry (ICP-MS) (Gao et al. 2015). Data were mean values from five independent biological replicates (five glasses) and each replicate contained five individuals.

### Transcriptome library preparation, sequencing, assembly and gene annotation

Transcription libraries were constructed using Illumina TruSeq™ RNA Sample Preparation Kit (Illumina, San Diego, USA), and cluster generation were performed using TruSeq PE Cluster Kit v3-cBot-HS (Illumina). The library preparations were then sequenced on an Illumina Hiseq 2000 platform and paired-end reads were generated.

After removing reads containing adapter and ploy-N, as well as low quality reads, clean reads were obtained. Their quality was also ensured via the assessment of Q20, Q30, GC-content and sequence duplication level. Transcriptome assembly was thus accomplished using Trinity (Grabherr et al. 2011).

All the assembled unigenes were searched against the following 7 database, including Nr (NCBI non-redundant protein sequences), Nt (NCBI non-redundant nucleotide sequences), Pfam (Protein family), KOG/COG (Clusters of Orthologous Groups of proteins), Swiss-Prot (A manually annotated and reviewed protein sequence database), KO (KEGG Ortholog database) and GO (Gene Ontology).

### Differential gene expression analysis

Gene expression levels were measured by RSEM (Li and Dewey 2011). For each sequence library, the read counts were adjusted by edge R program package through one scaling normalized factor. Differential expression of two samples was analyzed using the DEGseq (Anders and Huber 2010) R package. Q value was used to adjust P value (Storey and Tibshirani 2003). The threshold for significantly differential expression was q-value <0.005 & | log_2_ (fold change)| > 1.

### Enrichment analysis of DEGs

Gene ontology (GO) enrichment analysis of differential expressed genes (DEGs) was conducted by the GOseq R package according to Wallenius non-central hyper-geometric distribution (Young et al. 2010), where gene length was taken into account. A corrected P value of ≤0.05 was deemed as a threshold for significant enrichment of the genes. Top GO software was also applied to display enriched GO categories (Alexa and Rahnenfuhrer 2010).

KOBAS software was used to test the statistical enrichment of DEGs in KEGG (Kyoto Encyclopedia of Genes and Genomes) pathway to identify markedly enriched metabolic pathways or signal transduction pathways compared with the whole genome background. A corrected P value of ≤0.05 was considered as a threshold for significant enrichment of pathways in DEGs.

### Quantitative real-time PCR validation

Twenty genes were selected to carry out Quantitative real-time PCR (qRT-PCR) for the proving of DEGs results of Illumina sequencing. 1 μg RNA of CK and HAs were used to reverse-transcribe the first strand cDNA using the PrimeScript™ RT Reagent Kit (Takara). qRT-PCR was conducted on an optical 96-well plate with an iQ5 multicolor real time PCR system (Bio-RAD, USA). 20 μL reaction mixtures contained 1.0 μL of cDNA, 10 nM primers and 10 μL of iTaq™ Universal SYBR Green supermix (Bio-RAD, USA). The amplification conditions involved an initial step of 30 s at 95 °C, followed by 40 cycles of 10 s at 95 °C, and 20 s at 52 °C for annealing, and then 30 s at 72 °C for extension. For each sample, qPCR was repeated three times with the gene of *actin* (gene id: comp109453_c0) as endogenous control. Software CFX Manaer 2.1 was applied to calculate Cq value to analyze expressions of *actin* in CK and HAs. Relative expression level of specific gene was determined as described by Pfaffl (2001).

## Results

### Arsenic content and morphological comparison

After 14 days, seedlings under As stress treatment showed remarkable As accumulation in their roots, with mean content of 26.75 mg/kg, while mean level of arsenic of control (CK) was 0.79 mg/kg (Table 1). According to paired-samples *t* test, difference of As content between CK and HAs was very significant (*P* < 0.001) (Table 1). Arsenic content of 26.75 mg/kg also indicated *P. notoginseng* possesses the ability to over-accumulate arsenic.

**Table 1:** Paired-samples *t* test of arsenic content of CK and HAs^a^

	Mean (mg/kg)	N	Std.deviation	Std.error Mean	Sig.(2-tailed)
CK	0.794	25	0.086	0.017	
HAs	26.751	25	1.020	0.204	
CK-HAs	−25.957		1.036	0.207	<0.001

^a^ *HAs* seedlings of *Panax notoginseng* stressed by arsenic (40 mg L^−1^ Na_2_HAsO_4_·7H_2_O) for 14 days. *CK* seedlings for control

**Fig. 1:**
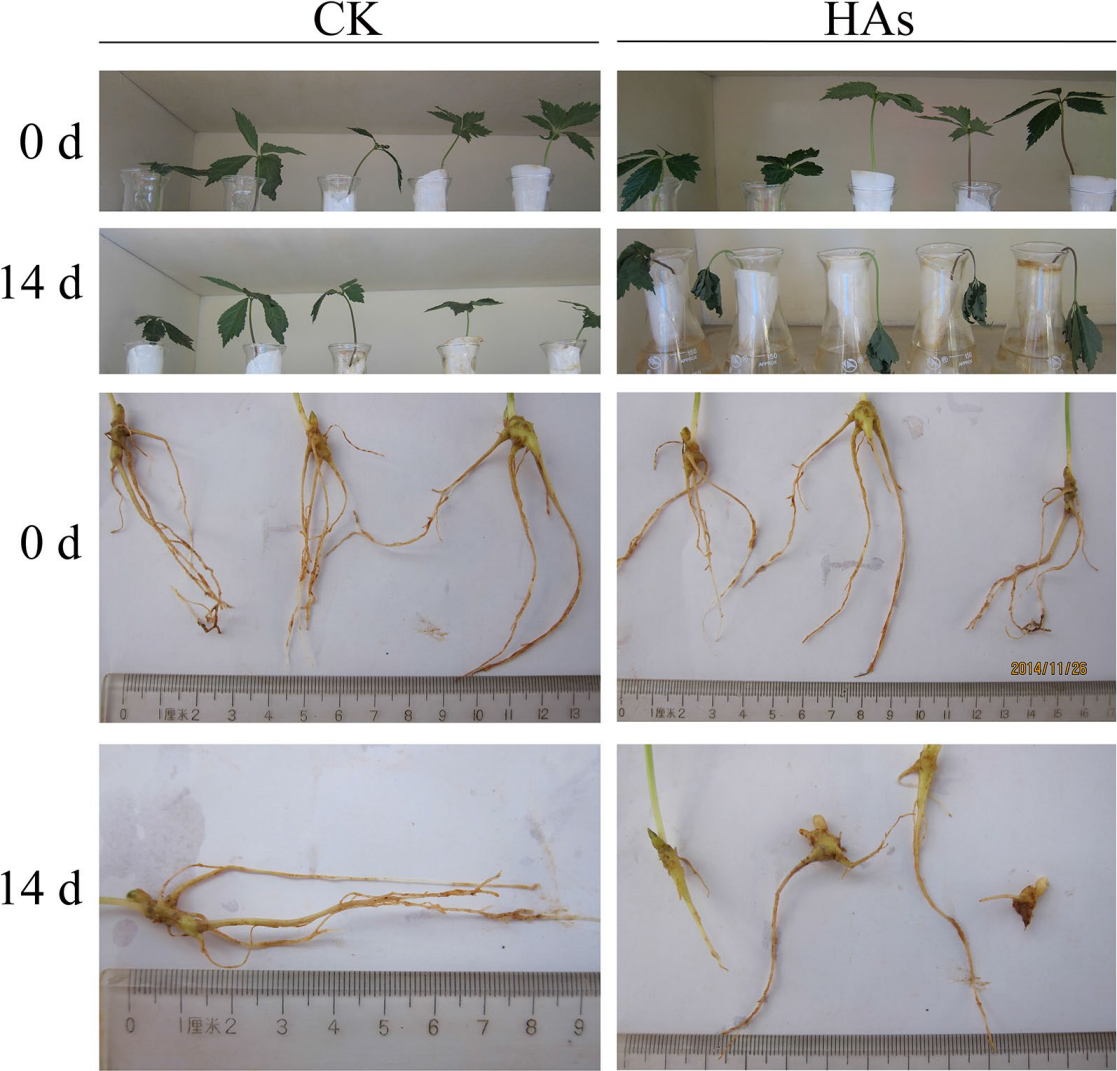
Morphological changes of seedlings of *Panax notoginseng* exposed to arsenic stress. *P. notoginseng* seedlings were stressed by arsenic (40 mg L^−1^ Na_2_HAsO4·7H2O) for 14 days (HAs). *CK* seedlings for control

Since changes of plant growth were considered as primary symptoms of As-toxicity, morphological traits were also compared between CK and HAs (Fig. 1). Obviously, plants of HAs were seriously dehydrated, their main roots got rotten and fibrous roots were almost absent (Fig. 1).

### Sequencing and assembly

After sequencing, there were totally 105,803,328 raw reads, 101,043,702 clean reads and 12.64 G clean bases in the two libraries. On average, 95.53 % bases of raw reads had a Q value ≥20 (the percentage of bases with a Phred value ≥20) and 91.39 % bases had a Q value ≥30 (the percentage of bases with a Phred value ≥30), with an error probability of 0.035 %. The GC-contents were 43.42 %.

308,350 transcripts were generated using Trinity software, with a mean length of 936 bp, N50 of 1719 bp and N90 of 345 bp. 172,355 unigenes were achieved, among which, 71.56 % unigenes (123,332) were 200–500 bp, 16.51 % (28,456) were 500–1 kbp, 7.81 % (13,467) were 1–2 kbp and 4.12 % (7100) were >2 kbp.

### Annotation and gene expression differences between HAs and CK

All of the 172,335 assembled unigenes were annotated in databases of Nr, Nt, Swiss-Prot, KO, PFAM, GO, KOG using the BLAST algorithm (E-value < 1E-5). There were 81,575 unigenes (47.34 %) annotated in at least one database, and 7867 unigenes (4.56 %) annotated in all databases. The number of unigenes annotated in databases of Nr, Nt, KO, PFAM, GO and KOG were 69,455 (40.29 %), 22,526 (13.06 %), 26,245 (15.22 %), 52,875 (30.67 %), 55,291 (32.07 %) and 32,507 (18.86 %), respectively. 47,287 (27.43 %) unigenes showed great similarity with unknown genes (hypothetical proteins).

Based on GO classification, 55,291 assembled unigenes were clustered into three functional categories: biological process, cellular component and molecular function. and further classified into 21, 14 and 11 subcategories respectively (Additional file 1: Figure S1). As for KOG functional classification, 32,507 matched unique sequences were classified into 26 categories (Additional file 1: Figure S2). 26,245 assembled unigenes were assigned to the following 5 KEGG biochemical pathways: metabolism (13,090 unigenes), genetic information processing (8976), organism system (7174), cellular processes (4106) and environmental information processing (3019) (Additional file 1: Figure S3).

**Fig. 2:**
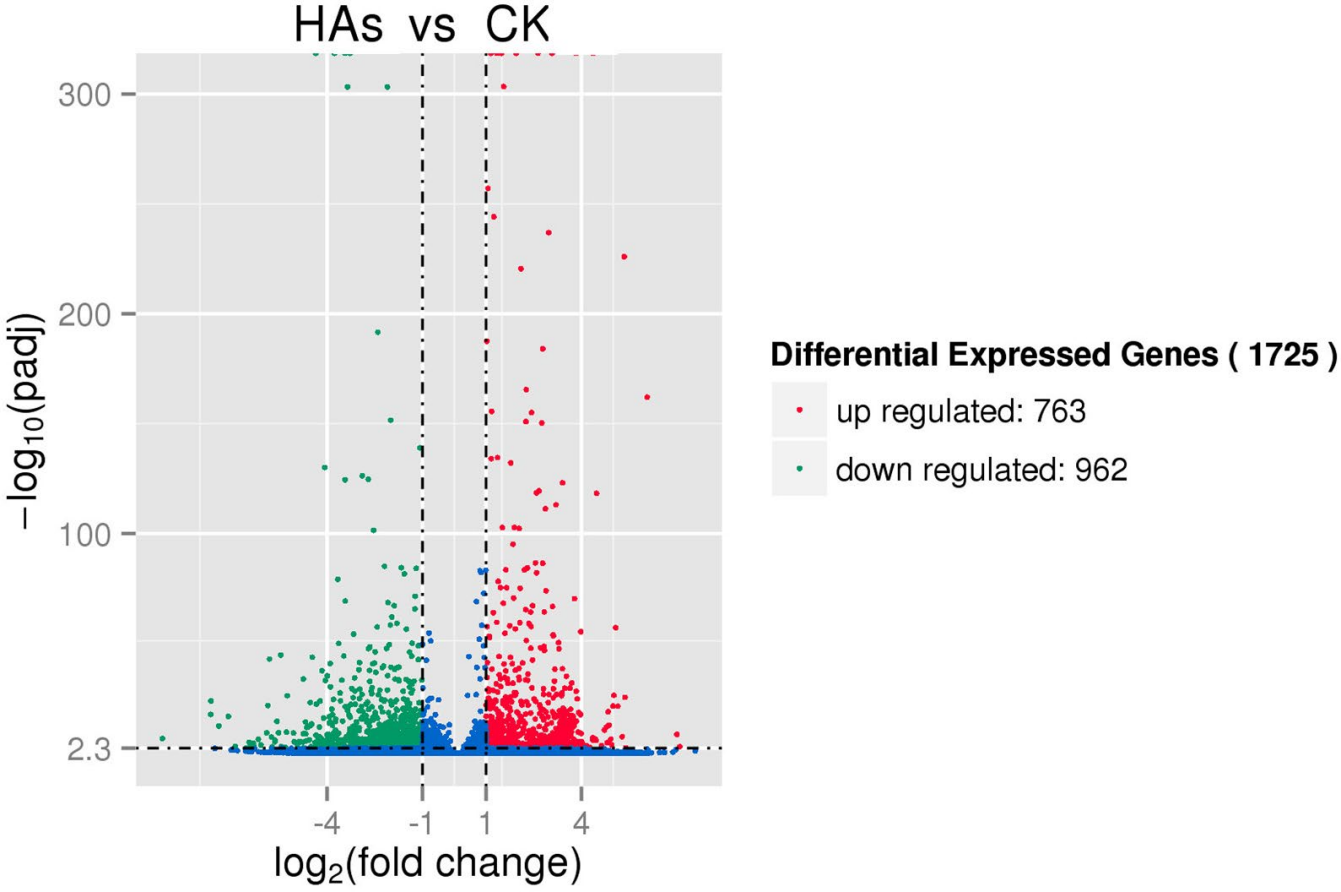
Expression patterns of differentially expressed genes (DEGs) identified between HAs and CK. *Red* and *green dots* represent DEGs, while *blue dots* are not DEGs. In total, 1725 unigenes were identified as DEGs (padj < 0.05) between HAs and CK, including 763 up-regulated genes and 962 down-regulated genes in CK. *HAs* seedlings of *Panax notoginseng* stressed by arsenic (40 mg L^−1^ Na_2_HAsO_4_·7H_2_O) for 14 days. *CK* seedlings for control

According to the criteria [q-value <0.005 and log_2_ (fold change) >1], 1725 genes (accounting for 1.00 % of total unigenes) were determined as significant DEGs between HAs and CK, including 763 up-regulated genes (44.23 % of significant DEGs) and 962 down-regulated genes (55.77 % of significant DEGs) in HAs (Fig. 2). The log_2_ fold varied from 1 to 9.18.

### Identification of genes involved in biosynthesis of ginsenosides and flavonoids

**Fig. 3:**
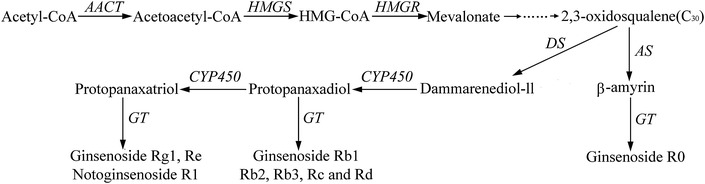
Putative ginsenoside biosynthesis in *Panax notoginseng*. *AACT* acetyl-CoA acetyltransferase, *HMGS* hydroxymethyl glutaryl CoA synthase, *HMGR* 3-hydroxy-3-methylglutaryl-CoA reductase, *AS* β-amyrin synthase, *DS* dammarenediol-II synthase, *CYP450* cytochrome P450, *GT* glycosyltransferase

**Fig. 4:**
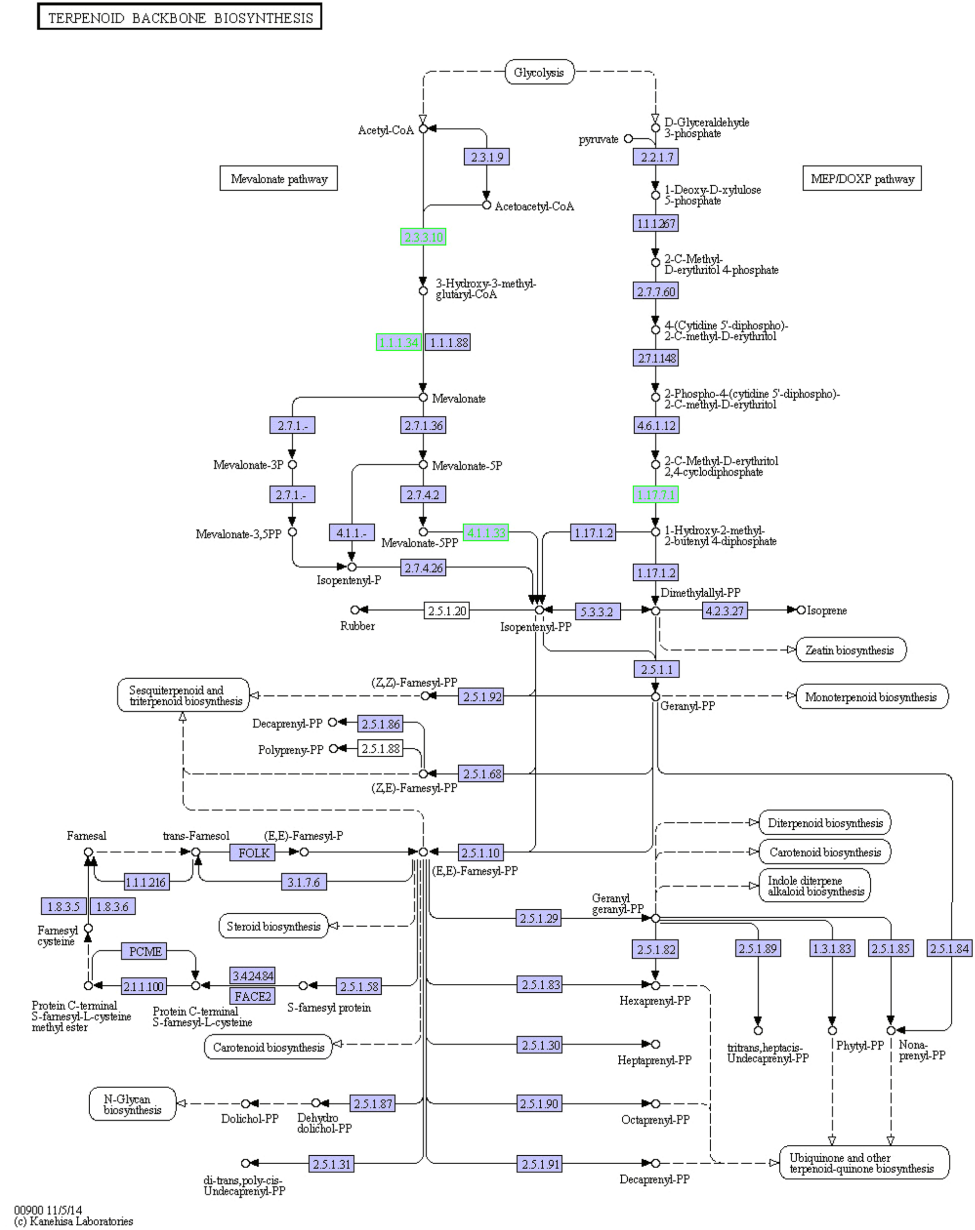
Unigenes predicted to be involved in the terpenoid backbone biosynthesis pathway. *Green* indicates genes with significantly decreasing expression; *white* indicates genes that were not identified in the expression profile analysis; *blue* indicates genes predicted to be involved in the pathway. *HAs* seedlings of *Panax notoginseng* stressed by arsenic (40 mg L^−1^ Na_2_HAsO_4_·7H_2_O) for 14 days. *CK* seedlings for control

Ginsenosides are synthesized by terpenoid backbone biosynthesis, followed by sesquiterpenoid and triterpenoid biosynthesis (Fig. 3). According to the putative pathway, totally 16 enzymes are involved in ginsenoside biosynthesis where two enzymes (CYP450 s and GTs) participate in the formation of various ginsenosides (Fig. 3). Among our 1725 DEGs, we identified 5 DEGs encoding enzymes involved in above pathway, namely 1 gene (comp129681_c0) of HMGS (hydroxymethyl glutaryl CoA synthase), 1 gene (comp106407_c0) of HMGR (3-hydroxy-3-methylglutaryl-coenzyme A reductase), 1 gene (comp140511_c0) of DS (Dammarenediol-II synthase) and 2 genes (comp126977_c0 and comp141084_c0) of GTs (glycosyltransferase) (Table 2). Of note, the 5 DEGs were all down regulated and mainly located in the beginning and the end of the pathway, i.e., arsenic stress may inhibit ginsenoside biosynthesis of *P*. *notoginseng* in the upstream and downstream steps. Such down-regulation was also shown in terpenoid backbone biosynthesis after the 1725 DEGs were likewise searched against KEGG database (Fig. 4).

**Table 2:** DEGs between HAs and CK identified by illumina and validated by qRT-PCR

Gene ID	Illumina sequencing	QRT-PCR	KO description
comp139796_c2	−53.83^a^	−138.82	chalcone synthase
comp78650_c0	−26.86	−80.31	flavonol synthase
comp129681_c0	−3.78	−9.73	hydroxymethyl glutaryl CoA synthase
comp106407_c0	−10.00	−25.62	3-hydroxy-3-methylglutaryl-coenzyme A reductase
comp140511_c0	−3.30	−5.73	Dammarenediol-II synthase
comp126977_c0	−6.04	−4.79	glycosyltransferase
comp141084_c0	−2.13	−6.34	glycosyltransferase
comp140248_c0	6.98^b^	19.41	glutathione S-transferase
comp117631_c0	8.19	25.85	glutathione S-transferase
comp128853_c0	2.05	63.56	myb proto-oncogene protein, plant
comp128641_c0	2.32	2.17	myb proto-oncogene protein, plant
comp66279_c0	3.55	2.63	myb proto-oncogene protein, plant
comp128496_c0	5.93	8.83	myb proto-oncogene protein, plant
comp77739_c0	8.52	8.26	myb proto-oncogene protein, plant
comp109390_c0	6.71	5.20	heat shock transcription factor, other eukaryote
comp141438_c1	5.22	9.27	heat shock 90 kDa protein
comp142366_c1	6.33	4.68	heat shock 70 kDa protein
comp141020_c0	7.02	21.39	heat shock 70 kDa protein
comp134360_c1	12.85	9.71	heat shock 70 kDa protein
comp134360_c0	13.58	20.37	heat shock 70 kDa protein

*HAs* seedlings of *Panax notoginseng* stressed by arsenic (40 mg L^−1^ Na_2_HAsO_4_·7H_2_O) for 14 days. *CK* seedlings for control. *DEGs* differentially expressed genes

^a^Negative values represent the folds of down-regulated expression of genes in HAs compared with CK

^b^Positive values represent the folds of up-regulated expression of genes in HAs compared with CK

**Fig. 5:**
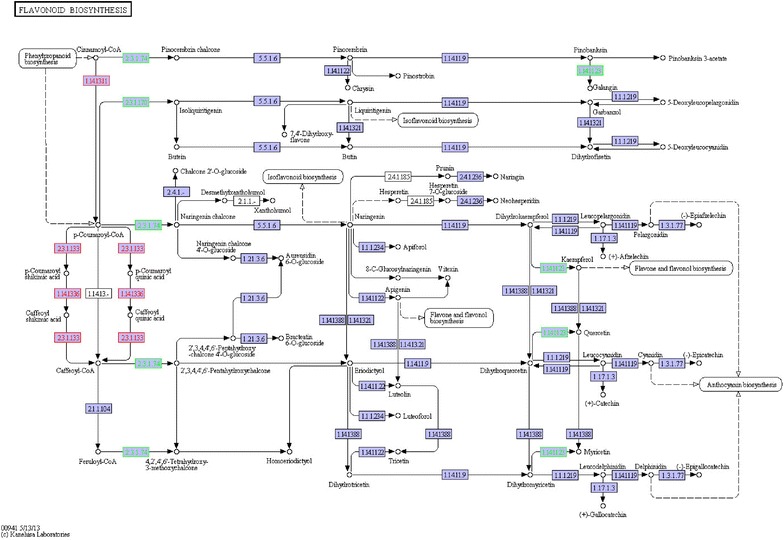
Unigenes predicted to be involved in the flavonoid biosynthesis pathway. *Red* indicates genes with significantly increasing expressions in HAs compared with CK; *green* indicates genes with significantly decreasing expression;* white* indicates genes that were not identified in the expression profile analysis; *blue* indicates genes predicted to be involved in the pathway. *HAs* seedlings of *Panax notoginseng* stressed by arsenic (40 mg·L^−1^ Na_2_HAsO_4_·7H_2_O) for 14 days. *CK* seedlings for control

Aside from ginsenosides, flavonoid biosynthesis was also depressed. The top three pathways with the most significant degree of DEGs enrichment in KEGG database were ‘starch and sucrose metabolism’, ‘plant hormone signal transduction’ and ‘phenylpropanoid biosynthesis’ with 29 DEGs, 25 DEGs and 20 DEGs respectively. As a branch of ‘phenylpropanoid biosynthesis’, ‘Flavonoid biosynthesis’ enriched 5 differentially expressed genes (Fig. 5). Among the 5 DEGs, 3 genes were up-regulated while the other 2 genes down-regultated. The 3 up-regulated genes were involved in the process of catalyzing Cinnamoyl-CoA to form Caffeoyl-CoA, while the 2 down-regulated genes can directly affect the ‘flavone and flavonol biosynthesis’ and ‘anthocyanin biosynthesis’. The 2 down-regulated genes were chalcone synthase (CHS, comp139796_c2, 53.84-fold lower in HAs) and flavonol synthase (FLS, comp78650_c0, 26.87-fold lower in HAs), which also located in the upstream and downstream of the pathway.

qPCR was applied to further validate the expression level of the above 7 down-regulated genes. Results indicated that, trends of expression differences of these genes detected by qPCR agreed with those concluded by Illumina sequencing (Table 2). Cq values of *actin* in CK and HAs were 24.66 and 26.93 respectively.

### Identification of genes related to tolerance mechanism of *P*. *notoginseng* to arsenic stress

As mentioned above, pathways of ‘starch and sucrose metabolism’ and ‘plant hormone signal transduction’ enriched the most DEGs in KEGG database. We also subjected genes up- and down-regulated with As exposure to GO analysis. Up-regulated genes were predominately involved in oxidoreductase activity, gene expression regulation (Additional file 1: Figure S4). Down-regulated genes mainly participated in carbohydrate metabolic process, polysaccharide metabolic process, cellulose metabolic process (Additional file 1: Figure S5). To further revealed the tolerance mechanism of *P*. *notoginseng*, especially processes of toxicity and detoxification, DEGs involved in arsenic accumulation, transporter, hormone, oxidative stress and transcriptional regulation were investigated.

Totally 7 genes related to arsenic accumulation were differentially expressed, including the up-regulation of 1 gene encoding hexose transporter (HXT) and 1 gene encoding natural resistance-associated macrophage protein (NRAMP), together with the down-regulation of 2 genes encoding Metallothionein (MT) and 3 genes encoding glutaredoxin (GRX). As for transporters, a number of genes encoding transporters were differentially expressed in HAs, including ATP-binding cassette (ABC) transporters, multidrug and toxic compound extrusion (MATE) transporters, as well as other transporters of sugar, oligopeptide and nitrate. As for hormone pathway, ethylene-related genes comprised the largest group of DEGs with the amount of 16 genes, and particularly, 12 ethylene-related genes were up-regulated with As exposure. Besides, DEGs related to hormone metabolism also involved ABA, cytokinin, et al. As for oxidation-reduction, 6 genes encoding thioredoxin and 3 genes encoding glutathione S-transferases (GSTs) were up-regulated in HAs. Besides, a great number of genes encoding transcription factors and molecular chaperones were also up-regulated with As exposure, including heat shock factors (HSF), Myb-related proteins (MYB) and heat shock proteins (HSP).

Based on qPCR verification, trends of expression differences of some above genes agreed with those obtained by Illumina sequencing (Table 2).

## Discussion

Root growth inhibition is the primary response of the plant exposed to arsenic and it is the As-sensitivity of the root that limits the productivity of the entire plant (Fu et al. 2014). Our study showed that, 14-day treatment of arsenic (40 mg L^−1^ Na_2_HAsO_4_·7H_2_O) could seriously damage the root growth of *P*. *notoginseng*, thus making the plants dehydrated.

Identification of genes involved in ginsenosides of *P*. *notoginseng* (Liu et al. 2015) provided us an insight to reveal the impact of arsenic accumulation to the quality of *P*. *notoginseng*. Our root transcriptomes showed that, As stress could reduce biosynthesis of ginsenoside and flavonoid by inhibiting gene expressions in the upstream and downstream steps of the pathways, indicating arsenic accumulation in *P*. *notoginseng* can cause qualitative losses, aside from the safety hazard reported previously (Liu et al. 2013a, 2010).

Flavonoid plays important roles in protecting organisms against biotic and abiotic stresses via eliminating reactive oxygen species (ROS) (Kumar et al. 2010; Liu et al. 2013b). Arsenic stress can induce the biosynthesis of flavonoid in *Sarcandra glabra* (Thunb) (Su and Zhou 2009), indicating flavonoid would be involved in positive response to As stress. However, our researches showed that, genes encoding chalcone synthase (CHS, comp139796_c2) and flavonol synthase (FLS, comp78650_c0), two key enzymes of flavonoid synthesis, were down-regulated in *P*. *notoginseng* under As stress. Such result contradicts previous findings obtained from other plant species and genus, but it corresponds well with an earlier report revealing the significant negative relationship between flavonoid content and As accumulation in *P*. *notoginseng* (Zu et al. 2014). Therefore, *P*. *notoginseng* may have different mechanism of As response, e.g., without resorting to flavonoid, and there may be other alternatives contributing to As response.

Tolerance mechanisms of plant to arsenic involve As accumuation and detoxification (Kumar et al. 2015). Therefore, genes related to As accumulation, transporter and hormone pathways may be As-tolerance associated (Fu et al. 2014; Kumar et al. 2015). In our study, genes encoding HXT and NRAMP were up-regulated and up-regulation of these genes can increase As accumulation (Shah et al. 2010; Tiwari et al. 2014), while genes encoding MT and GRX were down-regulated and up-regulation of these genes can lead to the decrease of As accumulation (Grispen et al. 2009; Sundaram et al. 2009). These results are good explanations to the ability of *P. notoginseng* to over-accumulate arsenic. Besides, genes encoding transporters (e.g., transporters of ABC, MATE, sugar, oligopeptide, nitrate, et al.) and genes related to hormone metabolism (ethylene, ABA, cytokinin) were differentially expressed, which also corresponded with those in *Arabidopsis* (Fu et al. 2014). Although *P*. *notoginseng* and *Arabidopsis* shared some common mechanisms in response to As stress, the quantity of As-tolerance associated genes (especially those involved in pathways mentioned above) was not as large as those revealed in *Arabidopsis*, and the degrees of expression differences of those DEGs were also smaller as a whole than those in *Arabidopsis* (Fu et al. 2014). Aside from genotype variation, treatment variation may also account for the differences. Compared with the research of *Arabidopsis*, duration of As exposure in our study was 14 days, much longer than 2 days. Therefore, it was the late stage of *P*. *notoginseng* to survive in As stressful condition that our transcriptional profiling revealed. The late stage can also be verified by the rotten roots and dehydrated plant of HAs. In this stage, As-tolerance associated genes may not as sensitive and active as those in early stage.

Glutathione S-transferases (GSTs) and thioredoxin are principally known for their role in detoxification reactions (Board and Menon 2013). Transgenic *Arabidopsis* plants over-expressing *OsGSTL2* (GST from rice) show an increase in tolerance to arsenic exposure (Kumar et al. 2013). In our study, up-regulation of GSTs and thioredoxin in HAs indicated GSTs and thioredoxin can develop plants with improved detoxification mechanism under As stress. Some isoforms of GST show dual activity, additionally functioning as a glutathione peroxidase in the presence of reactive oxygen species (Marrs 1996). Among the 3 up-regulated GSTs in our study, one belongs to Tau subfamily. The up-regulation of Tau class GSTs has also been noted in transcriptomic and proteomic analysis of plant roots under As stress (Norton et al. 2008; Ahsan et al. 2008). In addition, a great number of genes encoding transcription factors (TFs) and molecular chaperones, e.g. HSF, MYB and HSP, were also up-regulated under As treatment, indicating gene regulation at transcriptional level is the key mechanism of *P*. *notoginseng* to tolerate toxicity of As. The up-regulations of oxidative stress-related genes, TF genes and molecular chaperone genes in our study corresponded with those in *Arabidopsis* (Fu et al. 2014), indicating *P*. *notoginseng* and *Arabidopsis* take advantage of antioxidant and transcriptional regulation system to cope with the stressful condition.

MYB family and HSF family participate in various biological processes, including regulation of primary and secondary metabolism, defense and stress responses (Li et al. 2014, 2013c). There were multiple unigenes annotated to the same protein, which may represent different members of the same gene family. Plant HSFs are assigned to 3 classes (A, B and C) (Liu et al. 2013c). The up-regulated HsfB3 (comp109390_c0) in our study belongs to class B, which lacks AHA (aromatic, large hydrophobic, and acidic amino acid residues) in the activation domain. HSPs are known as molecular chaperones, essential for the survival of cells exposed to various stresses owing to their functions in folding, trafficking, maturation and degradation of proteins (Liu et al. 2013c). The 5 up-regulated HSPs in our study included 4 HSP70 and 1 HSP90. Consistent with our finding, arsenic can lead to up-regulation of HSP70 and HSP90 in *Labeo rohita* fingerlings (Banerjee et al. 2015), and expression of HSP70 can be induced by arsenic trioxide in MDA231 cells (Kim et al. 2005). Therefore, HSP70 and HSP90 would contribute to As response. Since HSPs are expressed under the regulation of HSFs at transcriptional level (Liu et al. 2013c), the above 5 up-regulated HSP genes might be supposed as target genes of HsfB3 under As stress in *P*. *notoginseng*. However, the underlying mechanism of the regulation is now an open question demanding further researches.

## Conclusions

Aside from safety hazard reported previously, our study found that arsenic accumulation can cause qualitative losses of roots of *P*. *notoginseng*, where biosynthesis of ginsenoside and flavonoid were depressed. Without resorting to flavonoid to fight against arsenic, *P*. *notoginseng* turned to oxidative stress-related proteins (GSTs, thioredoxin), transcription factors (HSFs, MYBs), molecular chaperones (HSPs), as well as transporter system (transporters of ABC, MATE, sugar, oligopeptide, nitrate) and hormone pathway (ethylene, ABA cytokinin) for survival. *P*. *notoginseng* and *Arabidopsis* shared some common mechanisms in response to As stress, but the quantity of related DEGs and the degrees of expression differences of specific DEGs were not as large as those in *Arabidopsis* (Fu et al. 2014). Aside from genotype variation, variation in duration of As exposure may also account for the differences.

## Additional file

**Additional file 1: Figure S1.** Gene Ontology classification of matched unigenes. 55,291 GO annotated unigenes were classified into 3 functional categories: biological process, cellular component and molecular function. **Figure S2.** KOG functional classification of matched unigenes. 32,507 KOG annotated unigenes were clustered into 26 categories. **Figure S3.** KEGG classification of assembled unigenes. 26,245 unigenes were assigned to 5 KEGG biochemical pathways: metabolism, genetic information processing, organism system, cellular processes and environmental information processing. **Figure S4.** Enriched GO terms of up-regulated genes. Genes up-regulated with As exposure were subjected to GO analysis. **Figure S5.** Enriched GO terms of down-regulated genes. Genes down-regulated with As exposure were subjected to GO analysis.
