# Supplementary material for: Illumina-based transcriptomic profiling of Panax notoginseng in response to arsenic stress
Source: Bot Stud. 2016 Jun 2;57:13. doi: 10.1186/s40529-016-0128-8 (PMC5432919; doi:10.1186/s40529-016-0128-8)
Supplement: Supplementary file 1 — Additional file 1: Figure S1. Gene Ontology classification of matched unigenes. 55,291 GO annotated unigenes were classified into 3 functional categories: biological process, cellular component and molecular function. Figure S2. KOG functional classification of matched unigenes. 32,507 KOG annotated unigenes were clustered into 26 categories. Figure S3. KEGG classification of assembled unigenes. 26,245 unigenes were assigned to 5 KEGG biochemical pathways: metabolism, genetic information processing, organism system, cellular processes and environmental information processing. Figure S4. Enriched GO terms of up-regulated genes. Genes up-regulated with As exposure were subjected to GO analysis. Figure S5. Enriched GO terms of down-regulated genes. Genes down-regulated with As exposure were subjected to GO analysis. [file 40529_2016_128_MOESM1_ESM.pdf]

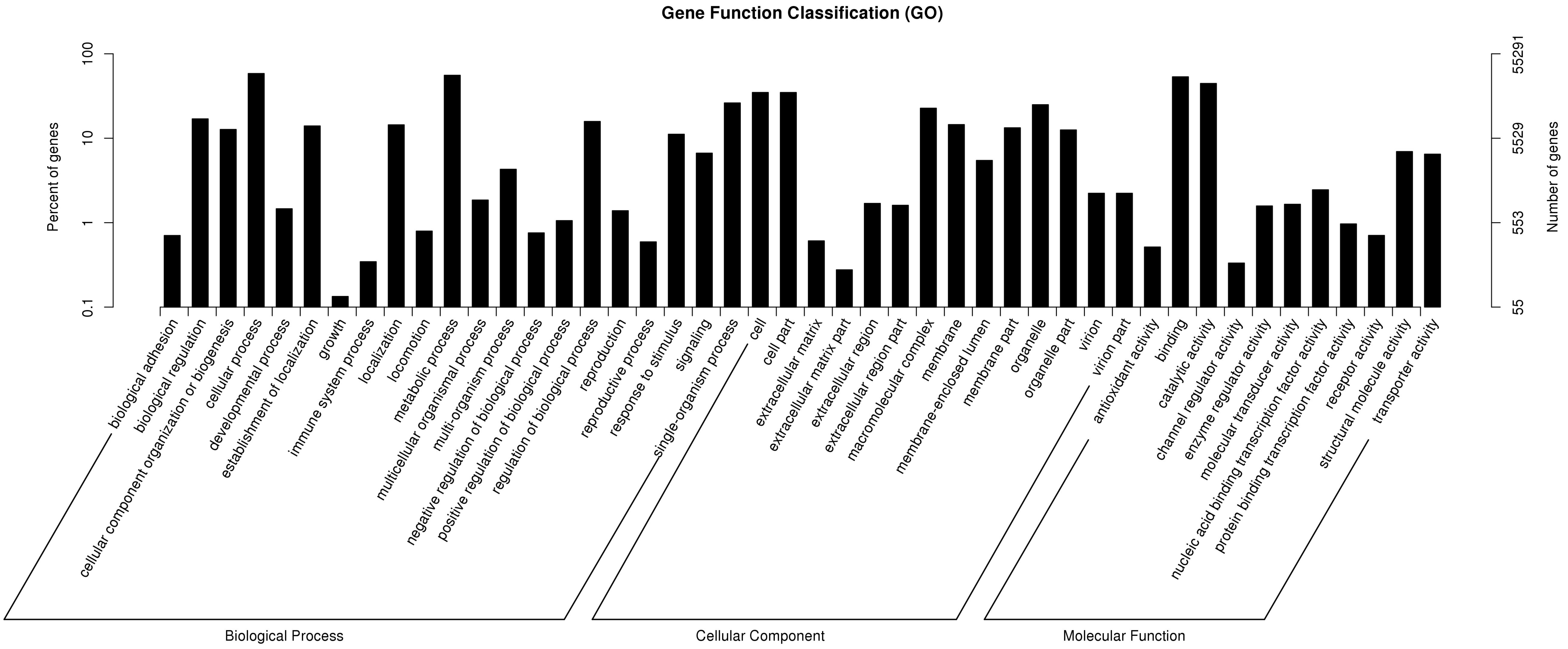

Figure S1. Gene Ontology classification of matched unigenes. 55,291 GO annotated unigenes were classified into 3 functional categories: biological process, cellular component and molecular function.

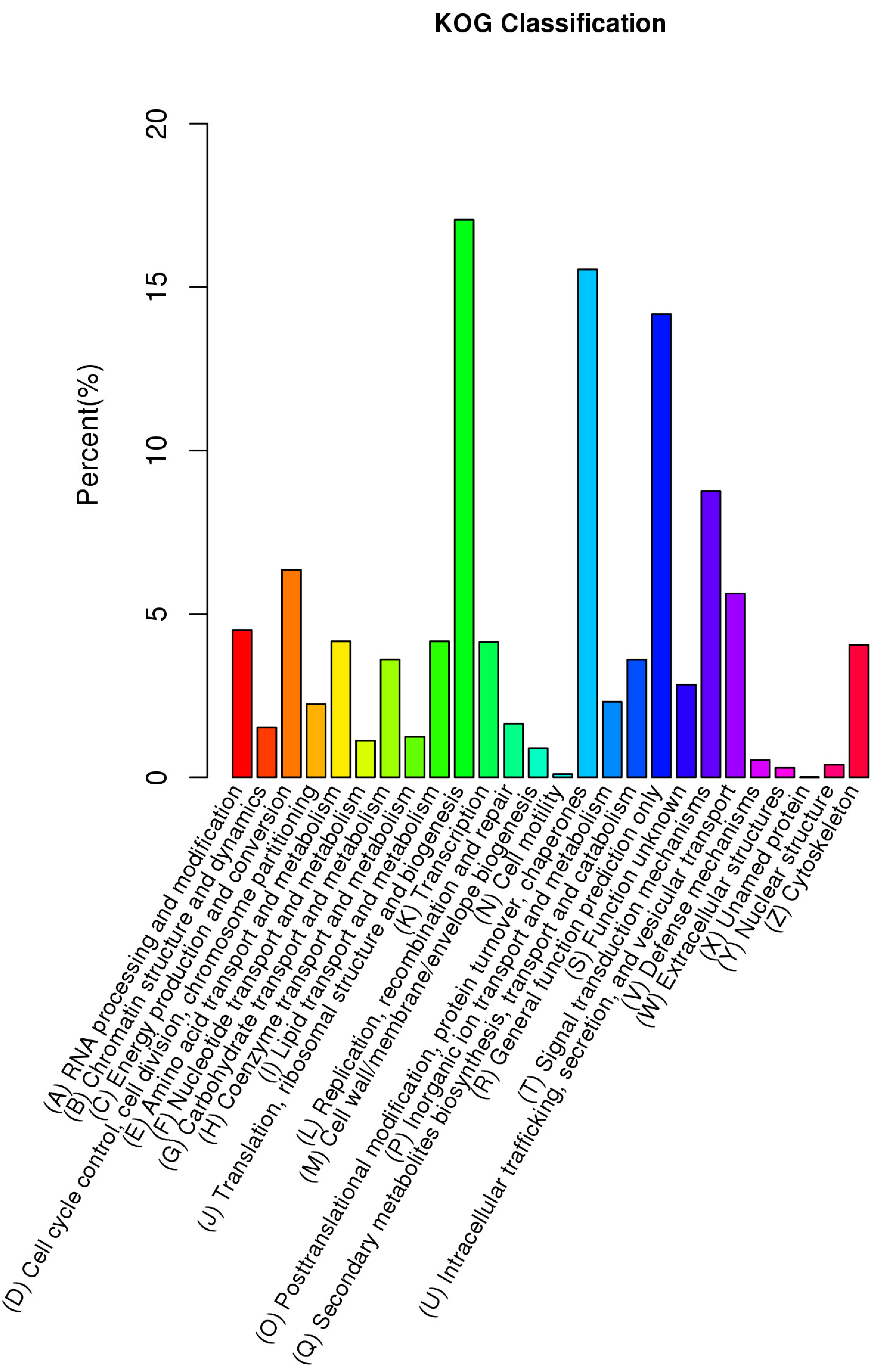

Figure S2. KOG functional classification of matched unigenes. 32,507 KOG annotated unigenes were clustered into 26 categories.

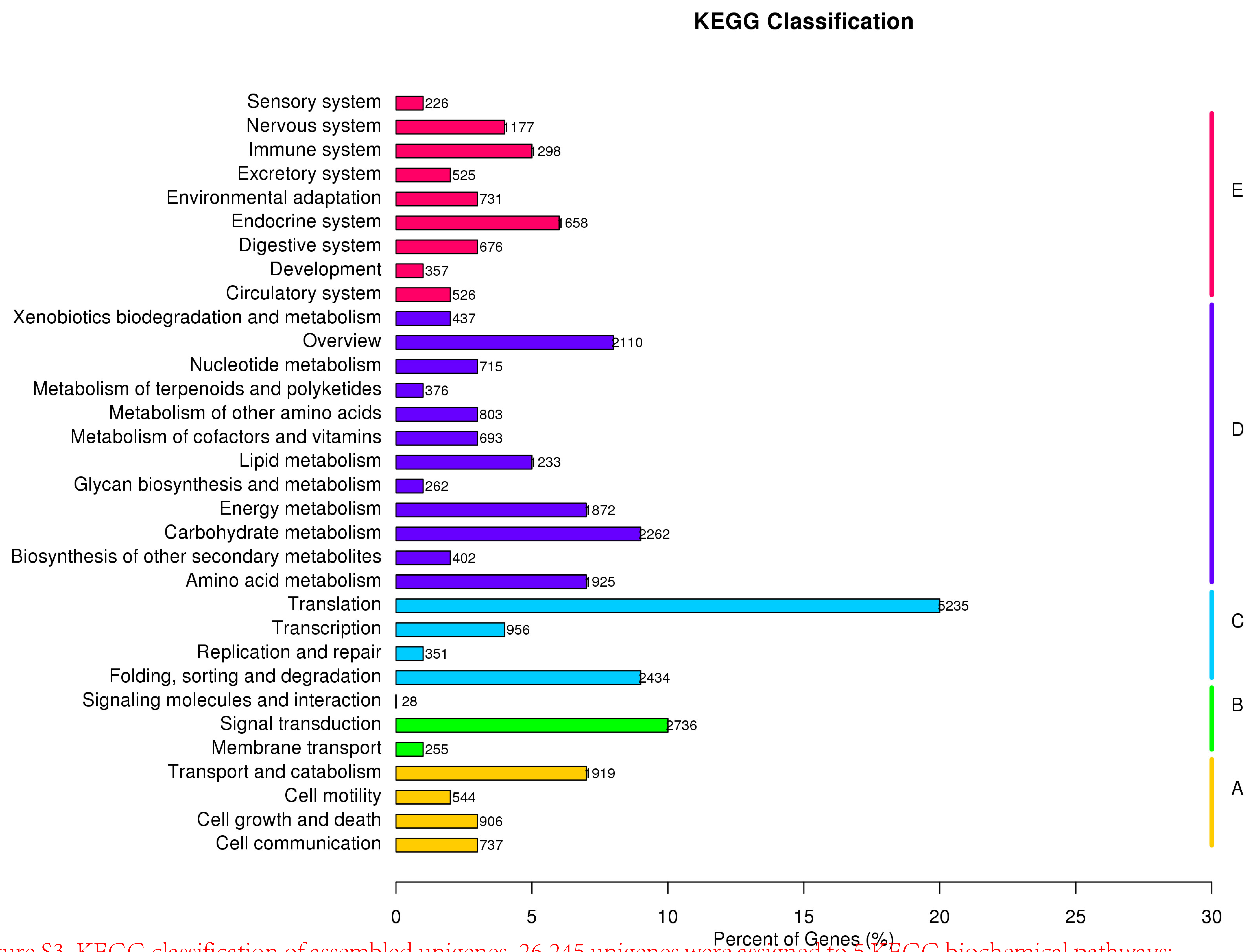

Figure S3. KEGG classification of assembled unigenes. 26,245 unigenes were assigned to 5 KEGG biochemical pathways: metabolism, genetic information processing, organism system, cellular processes and environmental information processing.

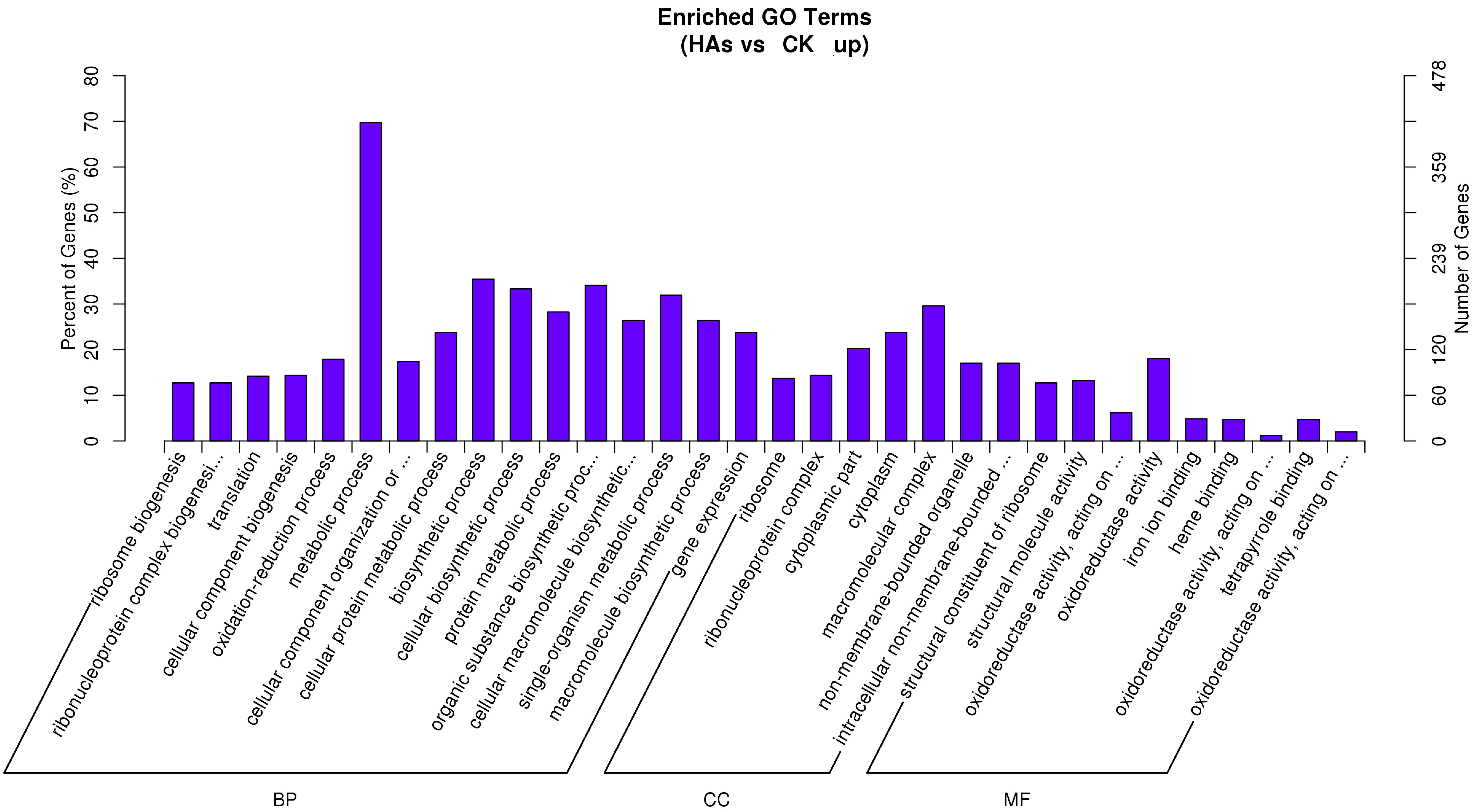

Figure S4. Enriched GO terms of up-regulated genes. Genes up-regulated with As exposure were subjected to GO analysis.

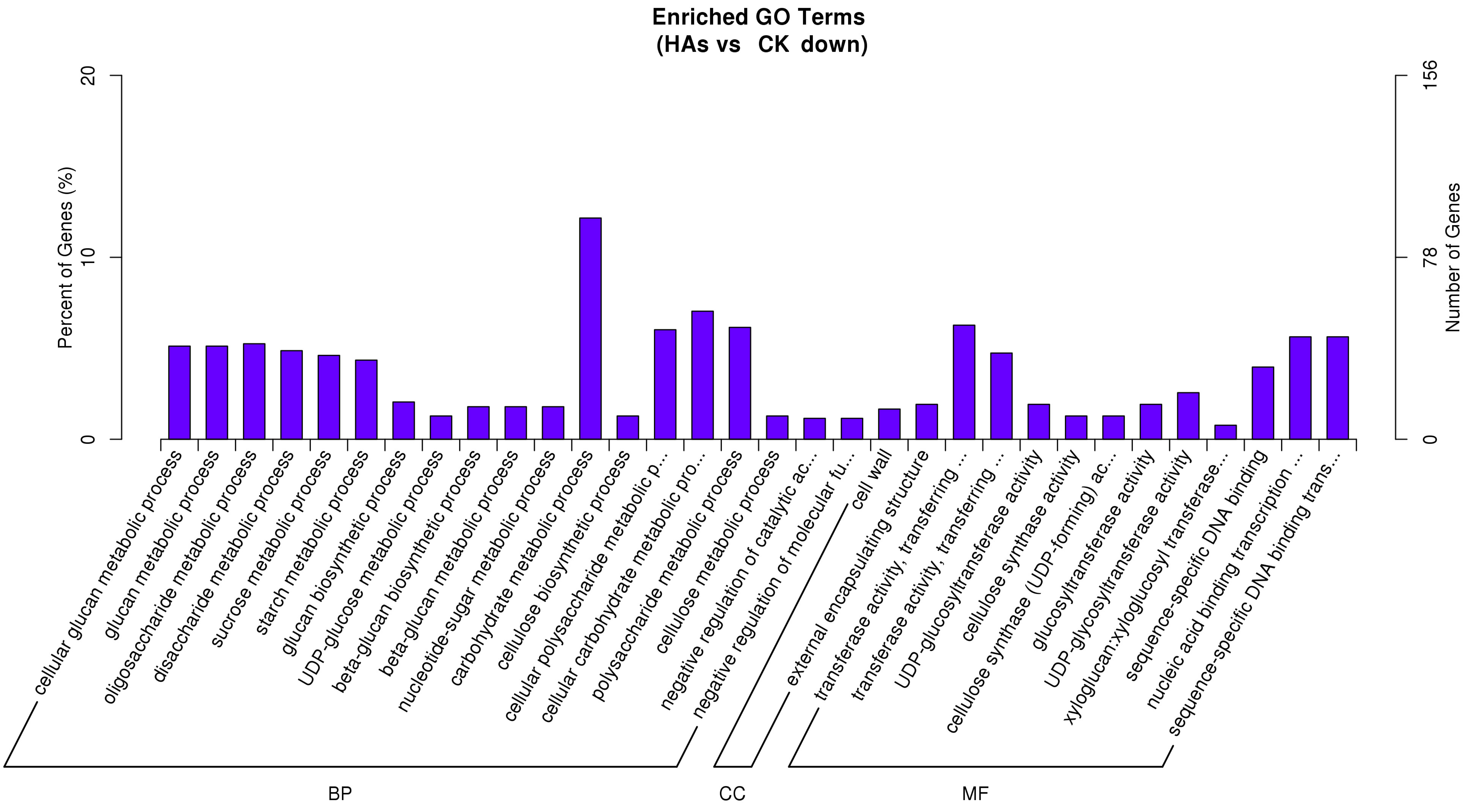

Figure S5. Enriched GO terms of down-regulated genes. Genes down-regulated with As exposure were subjected to GO analysis.
